# Gradual and Acute Temperature Rise Induces Crossing Endocrine, Metabolic, and Immunological Pathways in Maraena Whitefish (*Coregonus maraena*)

**DOI:** 10.3389/fgene.2018.00241

**Published:** 2018-07-19

**Authors:** Alexander Rebl, Marieke Verleih, Mareen Nipkow, Simone Altmann, Ralf Bochert, Tom Goldammer

**Affiliations:** ^1^Fish Genetics Unit, Institute of Genome Biology, Leibniz Institute for Farm Animal Biology (FBN), Dummerstorf, Germany; ^2^Research Station Aquaculture Born, Institute of Fisheries, Mecklenburg-Vorpommern Research Centre for Agriculture and Fisheries (LFA MV), Born, Germany

**Keywords:** aquaculture, DE genes, salmonid fish, transcriptome, temperature challenge, welfare

## Abstract

The complex and still poorly understood nature of thermoregulation in various fish species complicates the determination of the physiological status on the basis of diagnostic marker genes and indicative molecular pathways. The present study aimed to compare the physiological impacts of both gradual and acute temperature rise from 18 to 24°C on maraena whitefish in aquaculture. Microarray-based transcriptome profiles in the liver, spleen and kidney of heat-stressed maraena whitefish revealed the modulation of a significantly higher number of genes in those groups exposed to gradually rising temperatures compared with the acutely stressed groups, which might reflect early adaptation mechanisms. Moreover, we suggest a common set of 11 differentially expressed genes that indicate thermal stress induced by gradual or acute temperature rise in the three selected tissues. Besides the two pathways regulated in both data sets *unfolded protein response* and *aldosterone signaling in epithelial cells*, we identified unique tissue- and stress type-specific pathways reflecting the crossroads between signal transduction, metabolic and immunologic pathways to cope with thermal stress. In addition, comparing lists of differentially regulated genes with meta-analyzed published data sets revealed that “acute temperature rise”-responding genes that encode members of the HSP70, HSP90, and HSP40 families; their functional homologs; co-chaperones and stress-signal transducers are well-conserved across different species, tissues and/or cell types and experimental approaches.

## Introduction

The most economically significant fish species produced in the European Union and Norway are rainbow trout *Oncorhynchus mykiss* and Atlantic salmon *Salmo salar*. While these two salmonid species have been domesticated for decades (Tymchuk et al., 2009), the cultivation of the maraena whitefish *Coregonus maraena*, another salmonid fish, started only in the mid-1990s in Finland (Kause et al., 2011; Brietzke et al., 2016). Additionally, other European countries have established whitefish brood stocks for aquaculture industry (Arndt, 2001; Fopp-Bayat et al., 2015). Whitefish is a species-rich genus of mostly cold-water benthivores that grow optimally at water temperatures between 13 and 18°C (McCormick et al., 1971), while higher temperatures above 22°C impair growth (Jobling, 1981; Ficker et al., 2016) and temperatures above 26°C are lethal (Edsall et al., 1970; Edsall and Rottiers, 1976) for several coregonid species. According to a recently published mathematical model, increasing water temperatures due to global warming will reduce the biomass of wild whitefish populations by between 3 and 8% over a period of 50 years (Ficker et al., 2016).

Independent of global climate changes, farmed whitefish may occasionally or even persistently suffer from adverse housing conditions (Korytár et al., 2016). These include seasonally increased temperatures—closely linked to hypoxia—as whitefish have not yet or barely been selected for traits proven to be genetically correlated to stress resistance, as has been successfully carried out and documented for rainbow trout and Atlantic salmon (Pickering and Pottinger, 1989; Fevolden et al., 1991; Rexroad et al., 2013). Maraena whitefish is considered a highly stress-susceptible species (Arndt, 2001; Cingi et al., 2010; Korytár et al., 2016) and is thus a suitable model organism for studying physiological welfare aspects.

Alterations from thermal optima affect a wide range of molecular aspects: temperature-sensitive kinetics of enzymatic and non-enzymatic proteins are described, along with the temperature-dependent stability of nucleic acids and the saturation levels of either rigid or flexible cell membranes, as reviewed in Pörtner et al. (2007). These molecular-level perturbations are eventually decisive for the health and well-being of farmed fish, or may even make the difference between survival and death in aquaculture facilities lacking thermal refugia.

Heat-shock proteins (HSPs) are prominently investigated responders to different kinds of stress, primarily thermal stress. In recent years, however, research has not been limited to the physiological consequences of thermal stress to the response of HSPs and a pre-selected panel of related genes of interest. Instead, global approaches, such as microarray or RNAseq technologies, were utilized to study the mechanistic link between the environment and transcriptome and decipher comprehensive gene expression changes in salmonid fish species exposed to non-optimal or even lethal ambient temperatures. Such studies across different tissues and cell types proved on the one hand the common upregulation of apoptosis-related genes (Lewis et al., 2010; Rebl et al., 2013; Tomalty et al., 2015) and genes involved in innate immunity (Jeffries et al., 2012; Rebl et al., 2013; Tomalty et al., 2015; Verleih et al., 2015; Li et al., 2017), detoxification (Rebl et al., 2013; Li et al., 2017) and ion transport (Rebl et al., 2013; Tomalty et al., 2015) in addition to a diverse panel of induced stress-related genes (Vornanen et al., 2005; Lewis et al., 2010; Quinn et al., 2011a,b; Rebl et al., 2013; Anttila et al., 2014; Jeffries et al., 2014; Narum and Campbell, 2015; Tomalty et al., 2015; Verleih et al., 2015). On the other hand, genes involved in cold response (Jeffries et al., 2012; Rebl et al., 2013; Verleih et al., 2015) and mRNA translation (Jeffries et al., 2012) were found to be downregulated. Despite their differences, all these scientific reports document the conserved upregulation of particular genes, biofunctions and/or pathways in salmonid fish suffering from different kinds of thermal stress and contribute progressively to our understanding of thermoregulation and thus the well-being of farmed fish. Digital technologies will soon become prominent in farm animal production and high-throughput detection of biomarkers in farmed fish and ambient water are expected to regulate aquaculture husbandry conditions in optimal ranges in the near future.

The present study subjected the first generation of maraena whitefish under small-scale farming conditions to both gradual and acute rise in temperature from 18 to 24°C in order to record plasma parameters and differentially expressed (DE) genes to derive regulated biofunctions and pathways. These datasets were compared with meta-analyzed published datasets from different salmonid fish species to extract a common list of DE features with the potential to indicate temperature stress in salmonid aquaculture.

## Materials and methods

### Fish and experimental design

Maraena whitefish were bred from wild-fish eggs at the Institute of Fisheries, LFA M-V (Born, Germany), where the temperature experiments described below were conducted. Whitefish at the age of 50 weeks (349 days) post-hatch were randomly transferred from the recirculation system into nine 300-L indoor tanks at a density of ~30 kg/m^3^ for 7 d. Each of the nine tanks was identical (0.74 m length × 0.58 m width × 0.72 m height) receiving brackish water from the Darss-Zingst Bodden chain with 2.5–6 practical salinity units. Water was pretreated by drum filter and moving bed biofilm reactors in complement with UV radiation and exchanged about 0.5 times per hour. Monitoring characteristic parameters such as the concentrations of dissolved O_2_, ${\text{NH}}_{4}^{+}$, ${\text{NO}}_{2}^{-}$, ${\text{NO}}_{3}^{-}$, or pH in all tanks throughout the trial ensured a consistently high water quality. Maraena whitefish were fed commercial dry pellets by automatic feeders that distributed the food during the light period 12 h/d.

**Figure 1:**
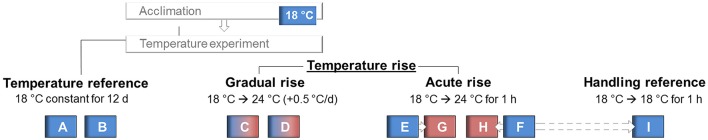
Experimental setup of the temperature experiment subjected to 50-week old maraena whitefish. Temperature in the “reference” tanks (labeled A, B, and I) was kept constant at 18°C throughout the experiment, while water temperature was increased up to 24°C in the “gradual temperature rise” tanks (labeled C, D). For the “acute temperature rise” experiments, maraena whitefish were transferred from 18°C tanks (labeled E, F) to 24°C tanks (labeled G, H).

Two of the nine experimental tanks (termed as A and B in Figure 1) were assigned as “temperature reference” tanks and kept at 18°C for the experimental period of 12 d. The water temperature in two other tanks (C and D), assigned as “gradual temperature rise” was elevated from 18 to 24°C by 0.5°C increments per day,. The remaining five tanks were used for “acute temperature rise.” Water temperature was kept constant at 18°C in two of these five tanks (tanks E and F) for 11 d, but on day 12, maraena whitefish were transferred from these tanks into 24°C-warm tanks (G and H), where the fish were kept for 1 h until sampling. To account for handling procedures during transfer, another fish group, assigned as “handling reference,” was transposed from an 18°C-tempered tank (F) to another 18°C tank (I), in parallel to those groups that were transferred to 24°C tanks (G and H). The handling and sampling procedures of maraena whitefish have been conducted in compliance with terms of the German Animal Welfare Act [§ 4(3) TierSchG] and approved by the institute's ethics commission. Throughout the experiment, no mortality or disease occurred among maraena whitefish and no technical problems were registered.

### Sampling and RNA preparation

At the end of the experiment, fish averaged 30.3 cm ± 1.7 cm in length (mean ± STD), and 300.6 ± 68.5 g in weight. Blood was sampled from the caudal vein of each whitefish using 5-ml plastic syringes filled with 500 μl 0.5 M EDTA (pH 8.0) solution. Tissue samples (entire liver; head and trunk kidney; entire spleen) were taken from seven maraena whitefish from each group.

Tissue fragments of liver, kidney and spleen were snap-frozen in liquid N_2_ and stored at −80°C until separate extraction of individual RNA with TRIzol reagent (Invitrogen, Carlsbad, CA, US). RNeasy Mini Kit (Qiagen, Hilden, Germany) in complement with the RNase-free DNase Set (Qiagen) gave high-quality RNA yields suitable for microarray hybridizations and subsequent quantitative real-time PCR (qPCR) assays.

Nucleic acid concentrations were quantified by measuring the 260:280 nm absorbance ratio with the NanoDrop 1000 spectrophotometer (NanoDrop Technologies/Thermo Fisher Scientific, Darmstadt, Germany). RNA was quality-checked with the Agilent 2100 Bioanalyzer platform (Agilent Technologies, Waldbronn, Germany) revealing RNA integrity number values ranging from 9.2 to 9.8.

### Plasma cortisol and glucose analyses

Blood samples of seven fish from each tank (A, B, C, D, G, H, and I) were centrifuged (4°C, 1700 rcf) and the supernatant was kept on ice until measurement of plasma parameters. According to the manufacturer's instructions, we analyzed the samples using a Cortisol ELISA Assay (DRG Instruments GmbH, Marburg, Germany) and the Glucose Assay Kit II (BioVision, Milpitas, California, US) with the Beckman Coulter DTX 800/880 Series Multimode Detector (Beckman Coulter, Brea, CA, US) for measuring absorbance at 450 nm.

### cRNA synthesis, array hybridization, and data analysis

Seven individual RNA samples from the same tissue (liver, kidney, spleen) and treatment (reference; gradual temperature rise; acute temperature rise) for each experimental replicate were proportionally pooled and labeled according to the One-Color Microarray-Based Gene Expression Analysis protocol, version 6.6. Briefly, 100 ng of each total RNA sample was amplified and labeled with the fluorescent dye cyanine 3 (Cy3) using the Agilent Low Input Quick Amp Labeling Kit (Agilent Technologies). The dye-incorporation of Cy3-labeled cRNA was measured with the ND-1000 spectrophotometer (NanoDrop Technologies/Thermo Fisher Scientific) revealing a rate between 12 and 20 fmoL/ng. Following fragmentation, 600 ng of Cy3-labeled cRNA was mixed with hybridization buffer (Agilent Gene Expression Hybridization Kit; Agilent Technologies) and hybridized to 8 × 60 K Agilent-049158 Salmon Oligo Microarrays (Agilent Technologies; Gene Expression Omnibus platform: GPL21057) at 65°C for 17 h. Microarrays were then washed once with the Agilent Gene Expression Wash Buffer 1 for 1 min at room temperature followed by a second wash with preheated Agilent Gene Expression Wash Buffer 2 at 37°C for 1 min.

The hybridized Agilent microarrays were scanned immediately after drying at 3-μm resolution (microarray scanner system G2505C; Agilent Technologies). The Agilent Feature Extraction Software (FES) 10.7.3.1 was used to read out the microarray image files. Extracted data were further analyzed using the limma package of the R version 3.1.1/Bioconductor suite (Smyth, 2005). Only genes with a corrected *p*-value of < 0.05 according to Benjamini and Hochberg (1995), and an absolute fold change of >2.0 were deemed DE genes and considered for further evaluation. Principal component analysis was done using the Rosetta Resolver Data Reduction (Weng et al., 2006). DE genes were re-annotated using the Basic Local Alignment Search Tool BLASTx. We considered only transcripts with unique BLAST results (coverage and sequence identity of >80% and *E*-value < 1 × 10^−4^) and joined redundant probes to prevent repeated counting of the same gene in subsequent analyses, such as comparisons of gene expression across different treatments using Venn Diagrams (Hulsen et al., 2008).

Annotated genes and corresponding fold-change (FC) values were imported into the Ingenuity program (Ingenuity Pathway Analyses/Qiagen) to evaluate the canonical pathways of interacting genes and visualize these in global functional networks. Benjamini–Hochberg multiple-testing was used for cut-off criterion. The Ingenuity Knowledge Base represents however a collection of experimental observations in mammalian species; therefore, we excluded the canonical pathways of mammalian diseases or exclusively mammalian pathways. Pathway enrichment analyses were complemented and cross-checked with DAVID-generated lists of KEGG pathways (Huang et al., 2009). In the following, predicted pathways and biofunctions are indicated by italic font. Standard scores (z-scores) were used to evaluate whether a particular pathway was activated (*z* > 1) or repressed (*z* < 1); for pathways with z-scores around 0, no prediction could be made.

### Confirmation of DE genes by fluidigm 48.48 real-time qPCR

Quantitative PCR was performed using the integrated microfluidic circuit technology of the Fluidigm Gene Expression chips (Fluidigm, Munich, Germany) and EvaGreen fluorescence dyes (Bio-Rad, Munich, Germany) to validate array-predicted mRNA abundances. The software Pyrosequencing Assay Design (version 1.0.6; Biotage, Uppsala, Sweden) suggested optimal whitefish-specific primers to amplify target fragments between 150 and 190 bp (Table 1). In addition, we used primer pairs that were originally designed for the amplification of gene fragments from trout and revealed a perfect conservation among rainbow trout and maraena whitefish (listed in Table 1). The resulting PCR products were ligated into a pGEM-T Easy Vector (Promega, Mannheim, Germany) and sequenced (Applied Biosystems 3130 Genetic Analyzer; Life Technologies) to verify the respective gene fragments.

**Table 1:** Gene-specific qPCR primers used in this study.

**Gene symbol**	**Sense (5′-3′)**	**Antisense (5′-3′)**	**Amplicon length (bp)**	**Primer efficiency (%)**
*AHSA1*	TGCCTCGATCCTTCATGTTCAG	GGAGACAAAGGAGGGAAGTTTC	157	89.2
*DDIT4a*	TAATCGCCTGTCTCTTTACGGG	TGCCAGAGTATGGAACAGATCG	157	101.2
*DNAJA4*	ATGGATCGCGAACTACTCCCAA	AAACGAAACATTCAGCGCAGCAT	154	102.3
*DUSP1*	AGAGGCATGGTGCTGTTTCAGA	AGAGCGCCAGAAGGTTTAGTTTA	158	99.9
*FOSL2*	ATAATAACCTCATTGGTCGAACTG	TACATTGATGTCAGGGATTTCTCT	174	100.0
HP^*^	GCACCCAGGCTTCCAGAACG	CAAGCCCCAGCCTGTGATGAT	169	101.1
*HSPA1Aa*	CTCCTCTGGGTTGAAGGATTTG	GGCCATGAACCCCAACAACAC	177	115.4
*PPP1R15A*	ACCCTTACCACCCTCTGAACG	GGAGGCTTTTGGCCACAACGT	162	98.3
*RGN*	ACGCCTATTTGCAGGTACCATG	TGAGGCTGTCTATGTAGTAGAAG	182	102.6
SERPINH1a^*^	ATGCATCGCACCGGTCTCTATA	ATCTTGCCCATCCAGGTTTCCA	181	102.5

* *Derived from orthologous sequences in Oncorhynchus mykiss*.

RNA samples (used previously for microarray hybridization) were individually reverse-transcribed into cDNA using the Reverse Transcription Master Mix (Fluidigm), preamplified using the PreAmp Master Mix (Fluidigm) and then treated with exonuclease I (New England BioLabs, Frankfurt/Main, Germany). All three steps followed the manufacturers' instructions. In the meantime, the primer assays were pipetted in duplicate into the 48.48 microfluidic circuits. Subsequently, the chip was primed in the MX IFC Controller (Fluidigm). Afterwards, the preamplified cDNA samples (*n* = 7 per tissue and treatment) were separately loaded on the primed 48.48 chip and analyzed with the Biomark HD system using the manufacturer's thermal protocol “GE Fast 48 × 48 PCR+Melt v2.pcl” (application type: gene expression; passive reference: ROX; assay: single probe).

Standard curves were performed for each primer pair using the LightCycler 96 System (Roche, Mannheim, Germany) in complement with the 2 × SensiFAST SYBR No-ROX Kit (Bioline, Luckenwalde, Germany) (Table 1). The qBase^+^ software (Biogazelle, Ghent University, Belgium) evaluated the suitability of a range of putative, whitefish-specific reference genes (see Altmann et al., 2015) based on averaged GeNorm stability values of *M* < 0.37 and coefficients of variation (CV) between 0.12 and 0.15. *RPL9, RPL32*, and *RPS20* were recorded as internal normalizer genes for liver samples, while *EEF1A1b, RPL9, RPL32*, and *RPS20* were used as normalizer genes for kidney and spleen samples.

Quantitative PCR data were analyzed using the Fluidigm Real-Time PCR Analysis software v. 4.0.1 and evaluated based on a linear regression of standard curves for each individual amplicon. Pearson's correlation test (http://www.socscistatistics.com/tests/pearson/) was used to assess the concordance between the microarray and qPCR results.

The full complement of microarray data was deposited in the NIH/NLM Gene Expression Omnibus (GEO, accession code: GSE95422), contributing to the initiative “Functional Annotation of All Salmonid Genomes” (Macqueen et al., 2017).

### Meta-analysis

The GEO database was scanned for gene expression studies focusing on gradual and acute temperature stress in salmonid fish. The gene lists of eligible microarray studies were searched for expression values of the respective target genes. The raw data from RNA-seq runs were received from the European Bioinformatics Institute database (EMBL-EBI) and reads were subsequently assembled to the respective target genes by using the Unipro UGENE bioinformatics software (Golosova et al., 2014). Total reads were counted and normalized to read counts of the reference genes *RPL9, RPL32*, and *RPS20*.

## Results

**Figure 2:**
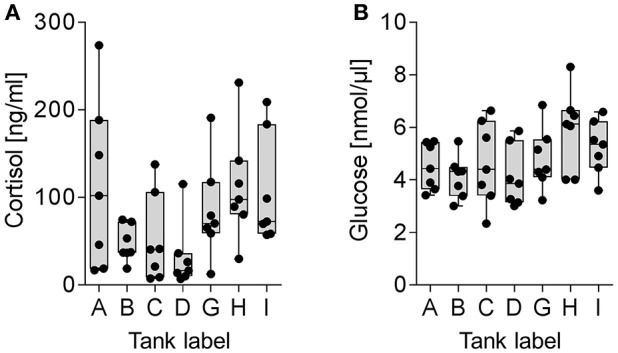
Plasma diagnostics of maraena whitefish kept in reference tanks (labeled A, B, I, abscissa), “gradual temperature rise” tanks (labeled C, D) and “acute temperature rise” tanks (labeled G, H). Box and whisker plots illustrate **(A)** cortisol levels (ng/ml) and **(B)** glucose levels (nmol/μl) in the plasma of seven individuals. Individual measuring points are indicated by black dots.

**Figure 3:**
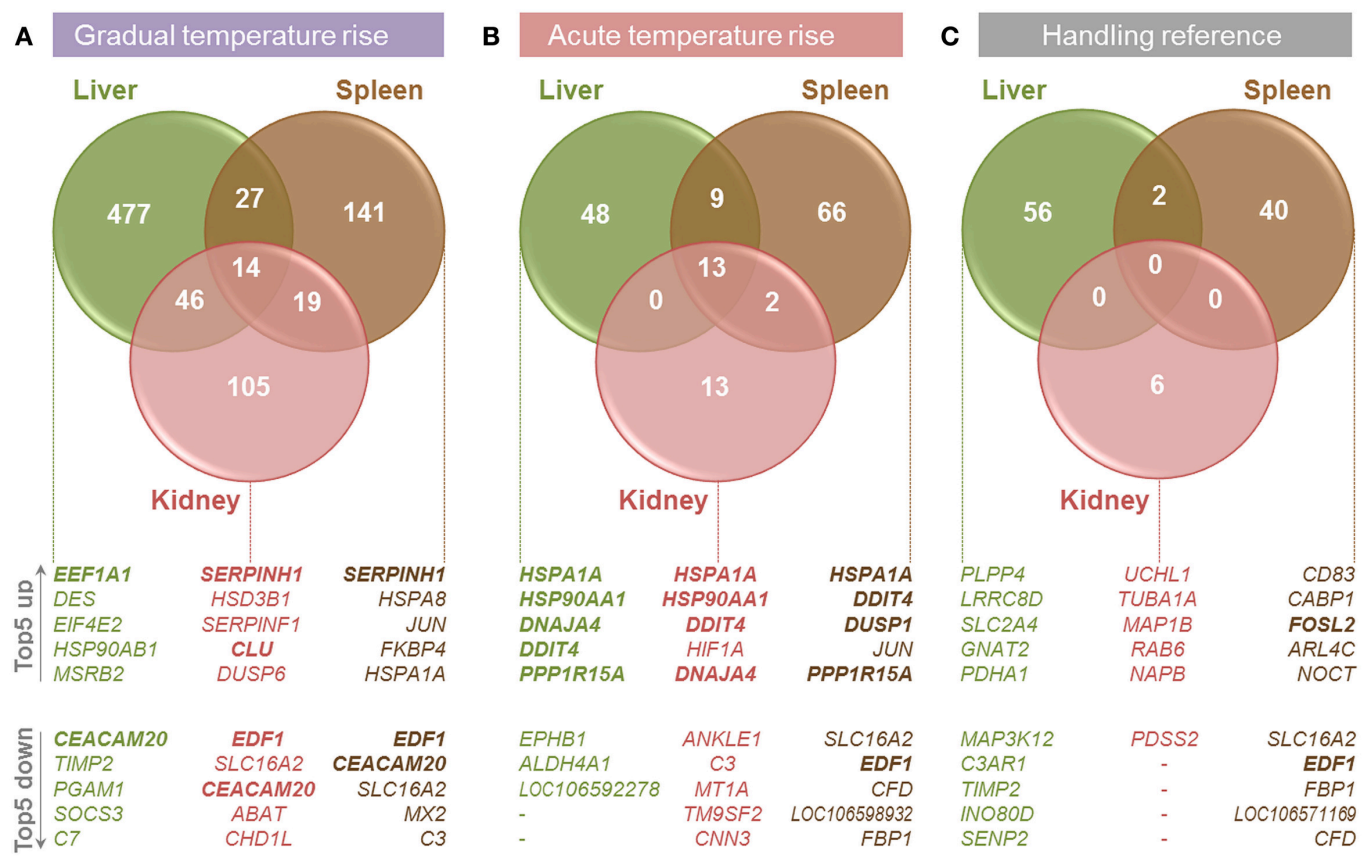
Overview of transcriptome analyses in the liver (green circles), spleen (brown), and kidney (red) of maraena whitefish exposed to **(A)** gradual temperature rise, **(B)** acute temperature rise and **(C)** handling relative to the data obtained for the “temperature reference” group. The number of DE genes is illustrated by Venn diagrams and the five most up- and down-regulated genes in the respective tissues are listed below. Commonly regulated features in all three tissues per treatment are printed in bold.

The present study compared the physiological impacts of gradual and acute temperature rise from 18to 24°C on maraena whitefish in aquaculture. Figure 1 provides an overview of the experimental setup. Although plasma cortisol (ranging from 7.0 to 273.7 ng/ml) and glucose levels (2.3–8.3 nmol/μl) did not show any significant differences between the analyzed groups due to high standard deviations (Figure 2), we recorded significant changes in gene expression in the challenged “gradual temperature rise” and “acute temperature rise” groups compared with the “temperature reference” group (Figure 3). The number of DE genes was 5-fold smaller in the “acute temperature rise” group compared with the number of DE genes in the “gradual rise” group (Supplementary Figures 1A,B). Moreover, the percentage contribution to the entirety of DE genes by the liver decreased in acute temperature rise (compared to gradual rise) on account of the spleen, while the percentage contribution by the kidney remained at a similar level.

### Tissue-specific responses to gradual temperature rise

In the liver of maraena whitefish, gradual temperature rise provoked the highest number of DE genes (*n* = 564, relative to the “temperature reference” group) compared with the spleen (*n* = 201) and kidney (*n* = 184) (Figure 3A). Diverse hepatic signaling pathways were predominantly attributed to blood cells (*B cell receptor, erythropoietin, GM-CSF signaling*, and *CD40 signaling*) (Table 2). In addition, the analysis predicted pathways directly (*ERK/MAPK signaling*) or indirectly (*ILK signaling, angiopoietin signaling*) involved in the response to stress.

**Table 2:** TOP5 list of functional pathways regulated in maraena whitefish exposed to gradual heat stress (18–24°C).

**Canonical pathway**	***p*-value**	**z-score**	**Involved DE genes^a^**
**SHARED BY LIVER, SPLEEN, KIDNEY**			
Unfolded protein response	<4.8E-04	n.a.	4–8 (54)
Acute-phase response signaling	<4.9E-03	−2.7 to 0.0	5–14 (169)
Gluconeogenesis	<1.1E-02	n.a.	2–5 (25)
Protein ubiquitination pathway	<2.0E-02	n.a.	5–10 (255)
Aldosterone signaling in epithelial cells	<2.3E-02	0.0 to 1.0	4–11 (166)
**SHARED BY LIVER AND SPLEEN**			
NRF2-mediated oxidative stress response	<2.7E-05	0.4	8–16 (193)
Glucocorticoid receptor signaling	<4.0E-04	n.a.	9–14 (287)
Interleukin-6 signaling	<8.0E-03	−1.5 to 0.0	4–11 (127)
JAK/Stat signaling	<1.5E-02	−0.7 to 0.0	3–8 (83)
Interleukin-17A signaling in fibroblasts	<2.0E-02	n.a.	2–6 (35)
**SHARED BY LIVER AND KIDNEY**			
EIF2 signaling	<3.2E-03	0.0 to 0.1	6–16 (220)
Superpathway of inositol phosphate compounds	<1.8E-02	n.a.	5–9 (230)
TR/RXR activation	<2.7E-02	n.a.	3–9 (98)
tRNA charging	<2.7E-02	n.a.	2–5 (39)
mTOR signaling	<4.2E-02	−0.3 to 0.0	4–12 (198)
**SHARED BY SPLEEN AND KIDNEY**			
Complement system	<1.8E-03	n.a.	3 (37)
FXR/RXR activation	<7.7E-03	n.a.	4–6 (126)
NADH repair	<2.0E-02	n.a.	1 (3)
Uracil degradation	<2.6E-02	n.a.	1 (4)
PRPP biosynthesis	<2.6E-02	n.a.	1 (4)
Thymine degradation	<2.6E-02	n.a.	1 (4)
**LIVER-SPECIFIC**			
B Cell Receptor signaling	3.3E-06	−0.8	14 (185)
Erythropoietin signaling	7.2E-06	n.a.	9 (79)
ERK/MAPK signaling	7.6E-06	0.0	14 (199)
ILK (integrin-linked kinase) signaling	3.0E-05	−0.1	13 (196)
GM-CSF (granulocyte-macrophage colony stimulating factor) signaling	3.1E-05	0.1	8 (73)
**SPLEEN-SPECIFIC**			
SAPK (stress-activated protein kinase) signaling	3.9E-03	−1.0	4 (104)
Phagosome formation	6.9E-03	n.a.	4 (122)
Eicosanoid signaling	8.2E-03	0.0	3 (67)
Leukocyte extravasation signaling	9.7E-03	−0.1	5 (210)
Lipid antigen presentation by CD1	1.1E-02	n.a.	2 (26)
**KIDNEY-SPECIFIC**			
Glutamate degradation	4.1E-04	n.a.	2 (5)
Aspartate degradation	8.6E-04	n.a.	2 (7)
Ubiquinol-10 biosynthesis	5.3E-03	0.0	2 (17)
GABA receptor signaling	9.5E-03	n.a.	13 (196)
β-alanine degradation	1.3E-02	n.a.	1 (2)

a *2.0 > FC < −2.0; corrected p < 0.05; the total number of pathway-involved genes is given in brackets. n.a., not available*.

In the spleen, gradual heat stress had an even larger impact on several immune pathways in leukocytes including lymphocytes and macrophages (*phagosome formation, eicosanoid signaling, leukocyte extravasation signaling, lipid antigen presentation by CD1*), whereas metabolic pathways were mainly found in the kidney (*glutamate, aspartate*, and β*-alanine degradation, ubiquinol-10 biosynthesis*) (Table 2).

### Tissue-specific responses to acute temperature rise

Acute temperature stress induced a significantly lower number of DE genes than did gradual temperature stress in the liver (*n* = 70, relative to “temperature reference”), spleen (*n* = 90), and kidney (*n* = 28) (Figure 3B). In the spleen, these regulated features were predicted to participate in the second messenger-triggered response to challenging stimuli (*cAMP-mediated signaling, G-protein coupled receptor signaling, protein kinase A signaling, ceramide signaling*) (Table 3). Also, in the liver and kidney, a general response to stress was reflected by indicative pathways, such as the stress-induced control of translation (*EIF2 signaling, regulation of eIF4 and p70S6K signaling*), stress signaling (*PCP pathway, mTOR signaling*), and metabolic responses (*glycolysis*) (Table 3).

**Table 3:** TOP5 list of functional pathways regulated in maraena whitefish exposed to acute heat stress (18–24°C).

**Canonical pathway**	**p-value**	**z-score**	**Involved DE genes^a^**
**SHARED BY LIVER, SPLEEN, KIDNEY**			
Unfolded protein response	<2.0E-05	n.a.	4–8 (54)
Aldosterone signaling in epithelial cells	<2.3E-06	n.a.	6–12 (166)
Glucocorticoid receptor signaling	<5.7E-03	n.a.	6–8 (287)
NRF2-mediated oxidative stress response	<2.5E-03	0.0 to 2.0	4–10 (193)
eNOS signaling	<1.6E-02	−1.0 to 0.0	3–5 (155)
**SHARED BY LIVER AND SPLEEN**			
Pyridoxal 5′-phosphate salvage pathway	<1.0E-02	n.a.	2–3 (65)
Salvage pathways of pyrimidine ribonucleotides	<2.1E-02	n.a.	2–3 (95)
PPAR signaling	<3.1E-02	n.a.	2 (93)
Cholecystokinin/gastrin-mediated signaling	<3.6E-02	n.a.	2 (101)
**SHARED BY LIVER AND KIDNEY**			
p53 signaling	<2.8E-02	n.a.	2–4 (111)
Aryl hydrocarbon receptor signaling	<4.3E-02	0.0	3–4 (140)
GADD45 signaling	<4.4E-02	n.a.	1–2 (19)
D-myo-inositol-tetrakisphosphate biosynthesis	<4.4E-02	n.a.	2–4 (141)
**SHARED BY SPLEEN AND KIDNEY**			
Urea cycle	<1.8E-02	n.a.	1–2 (6)
TR/RXR activation	<3.4E-02	n.a.	2–6 (98)
Stress-induced MAPK signaling	<3.7E-02	0.0 to 1.0	2–4 (102)
**LIVER-SPECIFIC**			
PPARα/RXRα activation	7.7E-04	0.0	4 (178)
Proline degradation	4.6E-03	n.a.	1 (2)
Glutamate biosynthesis/degradation	4.6E-03	n.a.	1 (2)
CD27 signaling in lymphocytes	6.5E-03	n.a.	2 (52)
PCP pathway	9.4E-03	n.a.	2 (63)
**SPLEEN-SPECIFIC**			
cAMP-mediated signaling	5.0E-04	−0.4	5 (223)
Regulation of Interleukin-2 expression in activated and anergic T lymphocytes	1.6E-03	n.a.	3 (79)
G-Protein coupled receptor signaling	8.4E-03	n.a.	4 (272)
Protein kinase A signaling	2.8E-02	0.0	4 (392)
Ceramide signaling	3.1E-02	n.a.	2 (93)
**KIDNEY-SPECIFIC**			
EIF2 signaling	2.3E-04	0.0	9 (220)
Glycolysis	1.6E-03	n.a.	3 (25)
mTOR signaling	2.6E-03	2.0	7 (198)
Regulation of eIF4 and p70S6K signaling	3.5E-03	n.a.	6 (156)
Cell cycle regulation by BTG family proteins	4.2E-03	n.a.	3 (35)

a *2.0 > FC < −2.0; corrected p < 0.05; the total number of pathway-involved genes is given in brackets. n.a., not available*.

### Tissue-specific responses to handling stress

As we expected that not only would the sudden temperature change affect gene expression in the “acute temperature rise” group, but also the transfer procedure itself, we analyzed the gene expression after transferring fish from one tank to another (Figure 3C). This handling stress had, as expected, a detectable impact on the mRNA levels of a particular number of genes in the liver (*n* = 58, relative to “temperature reference”), spleen (*n* = 42) and, to a lesser extent, kidney (*n* = 6). Of note, “gradual temperature rise” and “handling reference” shared 18 features in the liver and 22 features (including *EDF1*) in the spleen, while “acute temperature rise” and “handling reference” shared 15 features (including *DDIT4*) in the liver and 35 features (including *EDF1, PPP1R15A, FOSL2*) in the spleen. Regarding the kidney, there was no overlap between the DE gene lists among the respective groups.

### The responses to gradual and acute temperature rise are characterized by distinct gene-expression profiles

Pairwise comparisons revealed that a gradual temperature rise from 18 to 24°C induced the differential expression of a 2-fold (spleen) to 8-fold (liver) larger set of genes than did the acute temperature rise in the same range (Figures 3A,B and Supplementary Figure 1). In contrast, the lists of DE genes in fish transferred from the 18-°C tempered tanks to equally warm tanks (“handling reference”) or to tanks with 6°C warmer water (“acute temperature rise”) were similarly large (Figures 3B,C).

**Figure 4:**
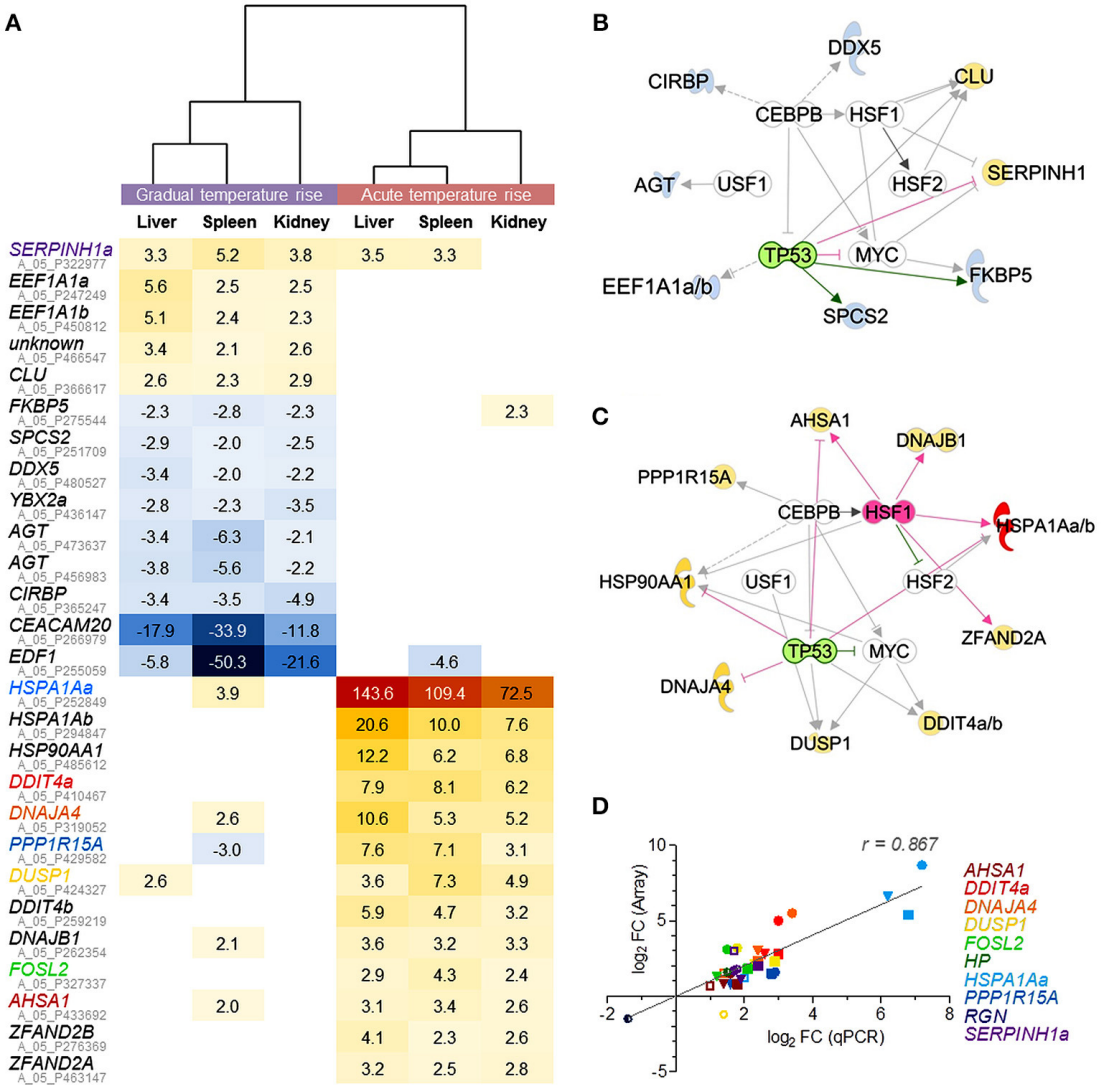
Microarray-based prediction of DE genes for gradual and acute temperature stress in maraena whitefish. **(A)** Hierarchical representation of DE features (*p* < 0.05; *q* < 0.05; −2.0 > FC < 2.0) shared by the liver, spleen, and kidney of maraena whitefish exposed to “gradual” (left panel) or “acute temperature rise” (right panel) as measured with microarray technology. The intensity and shade of the colored cells represent the normalized ratios, obtained after pairwise analysis with the “temperature reference group.” Gene symbols are listed with their respective Agilent IDs (in gray); the coloring of gene symbols (corresponding to the symbol colors in **D**) indicates that 10 selected FC values were validated using microfluidic qPCR. Interaction networks were generated using the IPA software to predict potential upstream regulators responsible for altered gene expression after **(B)** “gradual temperature rise” and **(C)** “acute temperature rise.” These regulators are arranged in the inner circle; predicted activation and inhibition are highlighted in pink and green, respectively. The affected genes are arranged in the outer circle; observed upregulation and downregulation are highlighted according to the colors of the heat map in **(A)**. The relationships between regulators and genes are displayed by arrows indicating induction (pink), repression (green) or uncertainty due to lack of knowledge or the state of downstream factors (gray). **(D)** The linear correlation plot shows the concordance of array (ordinate) and qPCR (abscissa) results for selected genes. Normalized log_2_ FC values were plotted for “gradual temperature rise” vs. “temperature reference” (open symbols), “acute temperature rise” vs. “temperature reference” (filled symbols) and “gradual” vs. “acute temperature rise” (semi-open symbols) comparisons in in the liver (circle), spleen (square) or kidney (triangle). Pearson's correlation is indicated. Symbol colors correspond with the colors of the gene symbols, as listed on the right margin and in **(A)**.

The comparison of DE gene sets in the liver, spleen, and kidney after gradual temperature rise pointed to 12 shared DE features (including duplicated *EEF1A1* copies and an unknown feature; *c.f*. Figure 4A, upper panel). This gene set includes both upregulated (*EEF1A1*) and downregulated features (*SPCS2, DDX5, EDF1*) linked with the biofunctions *regulation of transcription* and/or *translation*. Furthermore, it contains upregulated genes involved in *unfolded protein response* (*SERPINH1, CLU*) and downregulated genes related to *response to cold* (*YBX2a, CIRBP*) and *glucocorticoid receptor signaling* (*AGT, FKBP5*). Ingenuity bioinformatic analyses predicted that upstream transcription factor 1 (USF1) and the interaction of the transcriptional activators heat shock factors 1 and 2 (HSF1,−2), bHLH transcription factor MYC, CCAAT/enhancer binding protein beta (CEBPB), and deactivated tumor protein p53 (TP53) control the expression of the majority of the above genes, at least in mammals (Figure 4B).

A different set of 13 genes (including duplicated *HSPA1A* and *DDIT4* gene copies) was commonly upregulated across the liver, spleen and kidney after acute temperature rise (Figure 4A). Again, USF1, HSF2, MYC, CEBPB, and activated HSF1 with simultaneously inactivated TP53 were predicted to promote the expression of most of these 13 genes (Figure 4C) contributing to the pathways *aldosterone signaling* (*HSPA1Aa*, -*b, HSP90AA1, DNAJB1, DUSP1*) and *unfolded protein response* (*HSPA1Aa*, -*b, PPP1R15A, DNAJA4*), involving most likely co-chaperones (*AHSA1*) and factors with HSP-like functions (encoded by *ZFAND2A, ZFAND2B*), as well as factors regulating *cell proliferation* and *cell death* (*FOSL2, DDIT*) (Figure 4A, lower panel).

In conclusion, the upstream regulatory networks in Figures 4B,C suggest that an identical set of partially modulated transcriptional activators trigger very distinct responses to either gradual or acute temperature rise in the liver, spleen, and kidney. Although the two pathways *unfolded protein response* and *aldosterone signaling in epithelial cells* belong to the most-regulated pathways after gradual and acute temperature stress (Tables 2, 3), both are constituted by completely different gene sets. In addition, the two different treatment conditions activated several distinct stress type- and tissue-specific pathways.

### Microfluidic qPCR data confirm the microarray-identified expression differences of selected genes

The microfluidic qPCR technology was used to verify the array-predicted expression differences of ten selected genes induced by either gradual or acute temperature rise and that are known to be involved in different mammalian cellular pathways (*cf*. Tables 2, 3), i.e., *DDIT4a* (*mTOR signaling*), *DNAJA4* (*NRF2-mediated oxidative stress response*), *DUSP1* (*p38 MAPK signaling*), *HP* (*acute-phase response signaling*), *HSPA1Aa* (*aldosterone signaling in epithelial cell, unfolded protein response*), *PPP1R15A* (*EIF2 signaling*), *RGN* (*glucose/glucose-1-phosphate degradation*) and *SERPINH1a* (*collagen biosynthesis*), as well as *AHSA1* and *FOSL2* (Figure 4D). Profiles across these ten target genes confirmed a high concordance (Pearson product-moment correlation coefficient, *r* = 0.9) between both technologies. Moreover, qPCR data confirmed the statistical significance of the large majority of microarray-predicted FC values of fish exposed to the “gradual temperature rise” or “acute temperature rise” relative to the “temperature reference” fish (Supplementary Table 1).

### Meta-analysis endorses the modulation of particular gene-expression profiles upon acute temperature stress in different salmonid species

Our array analysis identified 22 genes (excluding duplicated copies) that were commonly regulated by gradual and acute temperature rise in the three investigated maraena whitefish tissues (*cf*. Figure 4A). Based on this list, we screened similar bioprojects having detected differentially regulated genes upon heat stress in salmonid fish species using microarray (Vornanen et al., 2005; Lewis et al., 2010; Quinn et al., 2011a,b; Rebl et al., 2013; Anttila et al., 2014; Jeffries et al., 2014; Verleih et al., 2015) or RNA-seq technique (Narum and Campbell, 2015; Tomalty et al., 2015). It should be noted that these investigations cover a broad temperature range (4–28°C) and also a broad range of tissues (including heart ventricle, kidney, gills, and blood cells), apart from different feeding regimes, sampling procedures and housing conditions.

**Figure 5:**
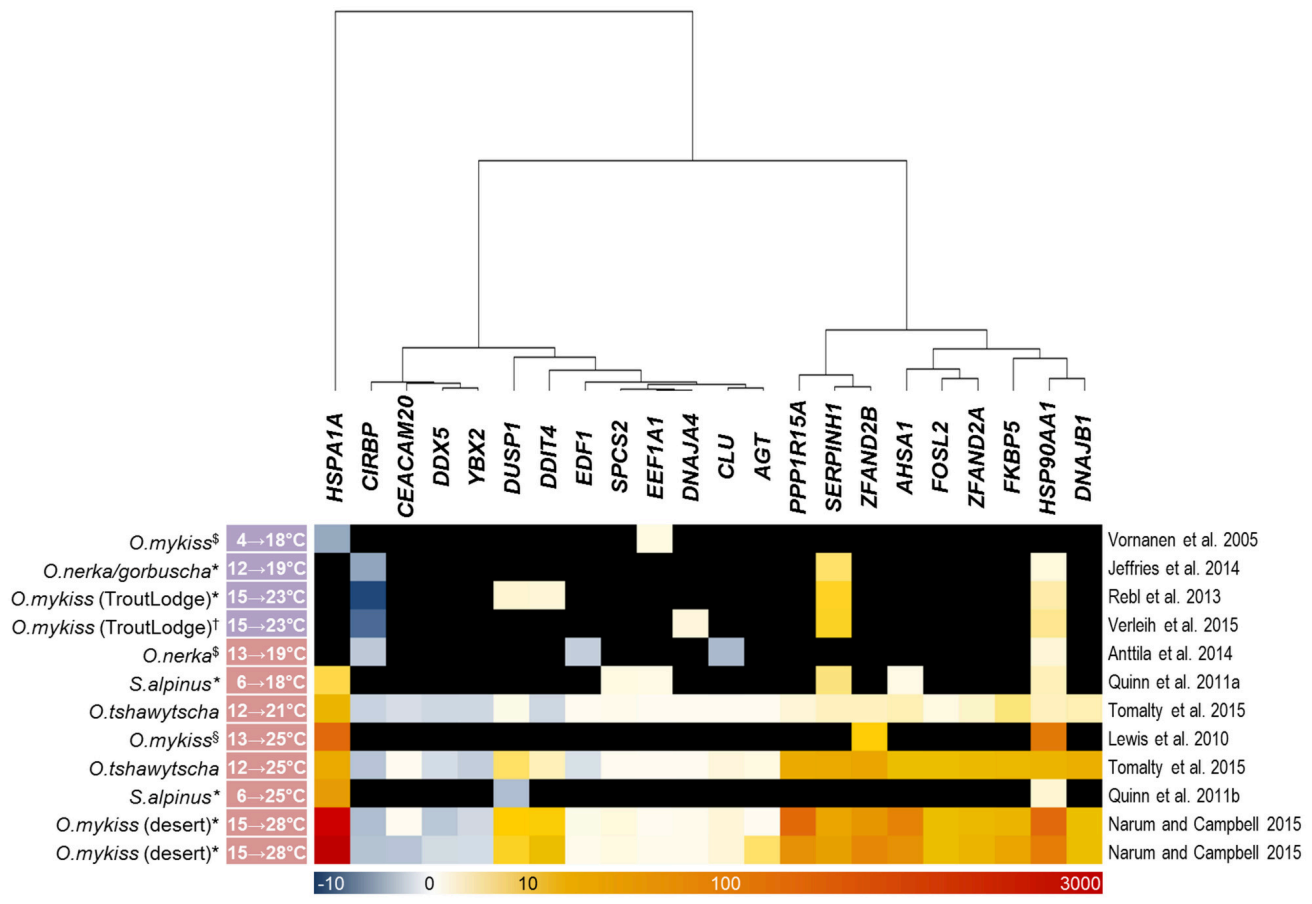
Thermoregulated genes in salmonid fish species after gradual and acute temperature rise (marked with a purple and red label, respectively, alongside the experimental temperature range given at the left-hand side) investigated in previous studies. The intensity and shade of the colored cells correspond to the color bar below the heat map and reflect FC expression values relative to the respective control groups; black fields denote missing information (only referring to microarray studies). Note that the included studies focused on different salmonid species (incl. strains) and—if applicable—tissues, marked with (^$^) for heart ventricle, (*) for gills, (^†^) for kidney, and (^§^) for red blood cells, as indicated. A literature reference is given at the right-hand side.

The enquiry confirmed, however, that the maraena-whitefish genes induced by acute temperature rise in our study have the potential to reliably indicate acute heat stress in salmonid fish in general, as they were also significantly upregulated in at least three out of the seven acute-stress studies (Figure 5). The only exception is *DNAJA4*, which was found to be upregulated by a factor of three in one single array-based investigation (Verleih et al., 2015). In contrast to acute-stress-responding genes, the genes responding to gradual stress revealed a rather occasional accordance with our list of DE genes from maraena whitefish. Nevertheless, we found for *SERPINH1, EEF1A1*, and *CIRBP* one to three gradual-stress studies supporting our results.

## Discussion

Improper husbandry conditions generally equate either to the sudden and possibly repeated appearance of one or even a combination of distinct environmental stressors (acute stress) or the persistent presence of stressful stimuli (chronic stress). Fish are—like all vertebrates—able to buffer exogenously induced perturbations to their homeostasis, which is reflected by various physiological alterations, including modulated gene expression. Therefore, particular gene-expression profiles indicate the type of stress exposure. The fast and reliable detection of an informative set of animal-based marker genes indicating the type and the severity of stress exposure would clearly improve welfare concepts and hence the health of farmed fish in aquaculture facilities.

In the present study, maraena whitefish were subjected to gradual or acute heat stress from 18 to 24°C. As expected, the majority of the commonly regulated genes in the investigated tissues encode cytoprotectors and key responders to stress, as discussed below. Moreover, we recorded the levels of the “traditional” plasma parameters cortisol and glucose, but we did not detect any statistically significant differences across the investigated groups. Previous analyses in salmonids revealed that cortisol concentrations of about 100 mg/ml and higher reflect stressful conditions (Procarione et al., 1999; Gesto et al., 2008; Conde-Sieira et al., 2010; Tahmasebi-Kohyani et al., 2012). We detected similarly high cortisol concentrations with high inter-individual variance in all groups analyzed. Since the maraena whitefish used in the present study were the first generation maintained in an anthropogenic environment and therefore considered as sensitive as wild fish, the elevated cortisol concentrations suggestively indicate the immediate response to the final sampling procedure rather than to the experimental stressor (see also Ortuño et al., 2002; Gräns et al., 2016). Additionally, numerous researches have expressed doubt about the reliability and information value of both parameters under certain circumstances (Martinez-Porchas et al., 2009; Roberts et al., 2010; Aerts et al., 2015; Rebl et al., 2017), also because preformed cortisol can be rapidly released upon exposition to an acute stressor, while gene expression changes take significantly longer periods of time (Das et al., 2018) and should thus be better suited to assess fish's long-term response to stress.

Both, gradual and acute temperature stress activated the expression of distinct—almost non-overlapping—sets of genes reflecting specific strategies either to adapt to slightly elevating ambient temperatures or to survive a heat shock. The repeated appearance of a low-intensity stressor was proven to eventually lead to attenuated stress responses. This desensitization process represents an important step on the way to the adaptation to moderately stressful conditions, as reviewed in von Borell (2000), and is characterized by comprehensive alterations in gene expression, as observed in this study for maraena whitefish within only 12 days in a gradually increasing ambient temperature. In contrast, harmful stress stimuli are likely to intensify the physiological response, known as sensitization. Provided that our temperature trial evoked two different responses in the direction of desensitization or sensitization, both responses were necessarily expected to be characterized by a cluster of particular DE genes.

### DE genes and specific pathways indicate the response to gradual temperature rise

The “gradual temperature stress” experiment was configured to mimic the seasonal elevation of water temperature in open aquaculture facilities. Surprisingly, we found downregulated features commonly present in liver, spleen, and kidney of gradually—but not acutely—stressed maraena whitefish. A conclusive explanation for this is that few of those genes are activated upon cooling, i.e., the cold-inducible RNA-binding protein (CIRBP), Y-box protein 2 (YBX2) and angiotensinogen (AGT).

CIRBP is probably the best characterized “cold-shock protein” in vertebrates (Phadtare et al., 1999). It is significantly upregulated after cold-shock (Gracey et al., 2004) and consequently down-regulated in fish during heat stress (Rebl et al., 2013; Verleih et al., 2015). Our research at gene-expression databases confirmed that reduced CIRBP levels are frequent indicators of gradual and also acute heat stress. For instance, in heat-stressed Chinook salmon *Oncorhynchus tshawytscha, CIRBP* has been downregulated together with *YBX2* (Tomalty et al., 2015). YBX2 (alias MSY2 and CSDA3) also belongs to the “cold-shock proteins” containing a “cold-shock conserved site” mediating the survival of prokaryotes at temperatures below the optimal growth temperature (Phadtare et al., 1999).

Low temperatures activate the renin–angiotensin–aldosterone system, of which angiotensinogen (*AGT*, alias *SERPINA8*) is a principal effector elevating blood pressure, thus reducing blood flow and heat emission (Hiramatsu et al., 1984; Cassis et al., 1998). *Vice versa*, the reduced *AGT* levels observed in gradually heat-stressed maraena whitefish most likely indicate accelerated blood flow to allow for increased oxygen transport rate in order to counteract hypoxia. Angiotensinogen is considered an acute-phase protein (Soden et al., 1994) that is involved in *acute-phase response signaling* in maraena whitefish exposed to gradual, but not acute, temperature rise. Here, AGT was predicted to act in conjunction with members of the complement cascade (C3, CFB), mitogen-activated protein kinases (MAPK), haptoglobin (HP) and other acute-phase proteins. The regulation of the *acute-phase response signaling* together with *complement system* during gentle temperature stress over a longer period of time has been previously observed in rainbow trout (Rebl et al., 2013).

The heat-shock protein HSP47, encoded by the *SERPINH1* gene, emerged in the present study as a clearly increased feature in different tissues of maraena whitefish challenged either by gradual or acute temperature rise. Previous studies into the thermal response of salmonids have demonstrated the significant potential of *SERPINH1* as a biomarker for heat stress in various tissues of different salmonid species, i.e., sockeye salmon *Oncorhynchus nerka* (Jeffries et al., 2012), rainbow trout *O. mykiss* (Rebl et al., 2013; Verleih et al., 2015; Wang et al., 2016; Li et al., 2017), Pacific salmon *O. gorbuscha* and *O. nerka* (Jeffries et al., 2014), Chinook salmon *O. tshawytscha* (Tomalty et al., 2015) and lake whitefish *Coregonus clupeaformis* (Stefanovic et al., 2016). HSP47 localizes to the endoplasmic reticulum (ER) and mainly chaperones collagen (Nagata et al., 1988; Nakai et al., 1992), a major component of the extracellular matrix, in mammals and in fish (Bhadra and Iovine, 2015). In this respect, it is worth noting that the predominant secondary structures in collagen are concatenated (hydroxyl-)proline residues and the related pathways *(hydroxyl-)proline degradation* were overexpressed in the liver of maraena whitefish that were challenged by gradual and acute temperature rise. Besides *SERPINH1*, we detected more upregulated members of the HSP family in the liver (*HSP90AB1, HSPB5, HSPA8*), spleen (*HSPA8, HSP1A1, HSP90AB1, DNAJA4, DNAJB1*) and kidney (*HSPA5*) of gradually stressed maraena whitefish, but these were not uniformly induced in all three tissues. We refer in this context to FK506-binding protein 5 (*FKBP5*), which inhibits the activity of the heterocomplex of HSP90 and the glucocorticoid receptor (Nair et al., 1997; Wochnik et al., 2005). *FKBP5* was downregulated in gradually stressed maraena whitefish indicating most likely a removal of barriers to enhance the responsiveness of the glucocorticoid receptor (Gillespie et al., 2009) reflected by the modulation of the *glucocorticoid receptor signaling* pathway in the liver and spleen. Interestingly, we and others (Narum and Campbell, 2015; Tomalty et al., 2015) found that acute stress provokes the upregulation of *FKBP5*, suggesting that the factor itself is tightly controlled dependent on the intensity, type and/or duration of stress.

Comparable to the *SERPINH1* mRNA abundance, the level of mRNA coding for clusterin (*CLU*) and the eukaryotic translation elongation factor 1-alpha 1 (*EEF1A1*, represented by duplicated gene copies) were significantly increased after gradual stress in maraena whitefish. CLU (alias *APOJ*) is not an HSP, but is considered as an extra- and intra-cellular chaperone that binds to misfolded proteins and inhibits their aggregation (Wyatt et al., 2009). Reflecting these vital functions, CLU has been referred to a “sensitive biosensor of environmental insults, specifically oxidative stress” (Trougakos, 2013) and “a protector against stress” in mammals (Criswell and Boothman, 2005). *EEF1A1* has been detected as an upregulated feature in heat-stressed rainbow trout (Vornanen et al., 2005). The encoded elongation factor supports the binding of heat-shock factor 1 to its response element in mammalian target promoters (Shamovsky et al., 2006) and ensures the fast export from the nucleus and the efficient translation of HSP1A1-encoding mRNAs (Vera et al., 2014). Moreover, EEF1A1 is inhibits the pro-apoptotic actions of TP53 (Blanch et al., 2013). This effect might have been enhanced by the down-regulation of *DDX5*, as observed in the present study in gradually stressed maraena whitefish. The DEAD-box helicase 5 is also termed p68 and since it acts as a co-activator of murine TP53 (Nicol et al., 2013) and is thus involved in multiple cellular decisions on survival and stress response in a context-dependent manner (reviewed in Shih and Lee, 2014).

Similarly, pleiotropic functions are attributed to the genes encoding endothelial differentiation-related factor 1 (*EDF1*), subunit 2 of the signal peptidase complex (*SPCS2*) and the carcinoembryonic antigen-related cell adhesion molecule 20 (*CEACAM20*), which were commonly repressed by gradual temperature rise in maraena whitefish. EDF1 (alias MBF1) interacts with calmodulin (Mariotti et al., 2004) and a member of the JUN family (Kabe et al., 1999) to suppress endothelial differentiation (Dragoni et al., 1998) and regulate a number of intracellular proteins, mainly signal transducers including those associated with the response to oxidative stress (Jindra et al., 2004). Members of the CEACAM family have manifold regulatory functions in epithelial, endothelial, and immune cells and it has been suggested that CEACAM20 transduces stimulatory signals (Zebhauser et al., 2005). The signal peptidase complex (SPC) is vital for the synthesis of all membrane and secretory proteins (Kalies and Hartmann, 1996). The downregulation of the subunit 2-encoding gene *SPCS2* in stressed maraena whitefish could indicate a generally reduced protein synthesis to leave energy resources for the dominant stress coping mechanisms. In this respect, the strong dependency on available glucose has been attributed to SPC subunit 2 besides its particular role during the *unfolded protein response* in mammalian cells (Hassler et al., 2015). In line with that, the *gluconeogenesis* pathway has been modulated as a prominent pathway in the liver, spleen, and kidney of gradually stressed maraena whitefish, but only in the kidney of acutely stressed maraena whitefish. The “neuroendocrine response to stress is characterized by excessive gluconeogenesis” (Marik and Bellomo, 2013), and it can be assumed that the duration of stress conditions enhance the hyperglycemia phenotype.

Altogether, we measured the dynamic regulation of almost two-hundred to more than five-hundred genes in the liver, spleen and kidney of maraena whitefish exposed to gradually rising temperature. This could strongly indicate the body's efforts to adapt to a moderate stressor. Despite the high number of DE genes, we identified only 11 genes that were both, up- and down-regulated—most likely in a systemic manner.

### DE genes and specific pathways indicate the response to acute temperature rise

Our “acute temperature rise” experiment exposed maraena whitefish to nearly critical temperatures, as proven for other coregonids (Edsall et al., 1970; Edsall and Rottiers, 1976) in terms of a “temperature shock.” Eleven genes showed pronounced inductions across the three investigated tissues of maraena whitefish. Our literature research confirmed that all these DE genes have been described earlier as highly regulated features after heat or hypoxic conditions.

The HSP70 family member *HSPA1Aa* was the most strongly upregulated gene of the present study and seems thus to be a biomarker candidate indicating a sudden “heat shock” in maraena whitefish. In fact, *HSPA1A* is the best investigated and most heat-inducible gene among vertebrates (Kampinga et al., 2009), including salmonid fish (Smith et al., 1999; Lewis et al., 2010; Quinn et al., 2011a; Narum and Campbell, 2015; Tomalty et al., 2015). A previous investigation on acute heat shock in Atlantic cod *Gadus morhua* (Hori et al., 2010) revealed that the mRNA abundance of *HSPA1A* increases by a level similar to that detected in the present study. The chaperone activity of HSPA proteins is generally regulated by DNAJ/HSP40 family members. In humans, the co-chaperone DNAJB1 is the most widely expressed and most heat-inducible member of the DNAJ family. In the three tissues investigated in maraena whitefish, *DNAJB1* was significantly upregulated together with *DNAJA4*. The latter gene is upregulated as early as 2 h after exposure to heat stress in chickens (Slawinska et al., 2016), a species with limited thermoregulatory capacities. Furthermore, *DNAJA4* is induced in the course of an *unfolded protein response* in Atlantic salmon (Bower and Johnston, 2010) and early after bacterial infection in channel catfish *Ictalurus punctatus* (Song et al., 2014). Though it has been documented that increased *DNAJA4* levels elevate the synthesis of cholesterol, a precursor of the stress hormone cortisol (Robichon et al., 2006), we detected only a slight tendency of higher cortisol concentrations in the “acute temperature rise” group compared to the “gradual temperature rise” group.

A second chaperone/co-chaperone pair that emerged as a strongly regulated features in the present study is *HSP90* and *AHSA1* encoding the activator of heat-shock 90-kDa protein ATPase homolog 1 (alias *AHA1*). *HSP90AA1* is already known as a universal biomarker indicating heat stress, in mammals and fish (Buckley et al., 2006; Quinn et al., 2011a,b; Anttila et al., 2014; Jeffries et al., 2014; Tomalty et al., 2015; Verleih et al., 2015). The ATPase activity of HSP90 is stimulated by AHSA1, which chaperones its own client proteins (Tripathi et al., 2014). In line with this, *AHSA1* has been identified as heat-shock responsive in Chinook salmon (Tomalty et al., 2015) and channel catfish (Liu et al., 2013).

Besides the canonical members of the HSP family, the two zinc finger AN1 type-containing protein-encoding genes *ZFAND2A* and -*2B* were upregulated in acutely stressed maraena whitefish. ZFAND2A (alias AIRAP) has been assessed as functionally homologous to canonical HSPs, as its functionality in humans is induced by heat and depends strictly on HSF1 (Rossi et al., 2010). The paralog ZFAND2B (alias AIRAPL) interacts with the proteasome, thereby stimulating the degradation of misfolded proteins (Braunstein et al., 2015). Moreover, ZFAND2A has already been suggested as a genetic marker in Atlantic cod *G. morhua* correlating with habitat differences in salinity and oxygen (Berg et al., 2015).

Our pathway analysis identified the *unfolded protein response* (the major ER stress pathway) and the *aldosterone signaling in epithelial cells* (the major pathway of electrolyte homeostasis) as two characteristic pathways for both acute and gradual heat stress in maraena whitefish. In the “acute temperature rise” group, both pathways were predominantly constituted by the *HSPA, HSPC*/*HSP90* and *DNAJ* members of the diverse HSP family and this gene set overlaps only marginally with the respective one of the “gradual temperature rise” group. In this respect, we refer to the gene coding for the regulatory subunit 15A of protein phosphatase 1 (*PPP1R15A*, alias *GADD34*) that was upregulated in acutely stressed trout (Narum and Campbell, 2015; Tomalty et al., 2015) and maraena whitefish in the current investigation. The stress-inducible PPP1R15A (Fornace et al., 1988) supports the cellular recovery from the *unfolded protein response*, as suggested for mouse (Kojima et al., 2003) and grouper *Epinephelus* spp. (Lu et al., 2012). The pathway analysis revealed additionally that *NRF2 (Nuclear factor erythroid 2-related factor 2)-mediated oxidative stress response* together with the *eNOS signaling* pathway was activated in all three investigated tissues of acutely stressed maraena whitefish, possibly mirroring the increased utilization of oxygen accompanied by elevated rates of reactive oxygen species in cold-water fish.

The immediate-early gene *FOSL2* (alias *FRA2*) encodes the leucine zipper-containing FOS-like antigen 2 heterodimerizing with proteins of the JUN family, which are upregulated together with *FOSL2* by “acute temperature rise” (*cf*. panel “TOP5 up” in Figures 3C,D). FOSL2 and JUN form the transcription factor AP-1, which in turn regulates cell proliferation, differentiation, and transformation. *Cell proliferation* is among the regulated pathways in brown trout *Salmo trutta* suffering from oxidative stress with *FOSL2* as one of the most strongly upregulated features (Uren Webster and Santos, 2015). Stress experiments in mice have disclosed that mRNA levels of *FOSL2* recover fast (Weinberg et al., 2007) suggesting that this gene might be indeed an informative indicator of acute stress responses. The experimental exposition of zebrafish to acute oxidative stress induced a series of genes involved in stress response, including *FOS* together with *HSP1A1, HSP90AA1*, and *DNAJB1* (Hahn et al., 2014). In that study, *DUSP1* (alias *MKP1, PTPN10*) also emerged as a fast responsive gene. The encoded dual specificity phosphatase 1 is a short-lived protein (Brondello et al., 1999) that dephosphorylates the mitogen-activated protein kinases ERK, JNK, and p38, and is thus intimately involved in stress signal transduction and, most likely, also in acutely stressed maraena whitefish.

The DNA damage-inducible transcript 4 *DDIT4* (alias *REDD-1* and *Rtp801*) is another factor involved in stress signal transduction. *DDIT4* has been identified as a heat- and hypoxia-responsive target gene of the hypoxia-inducible factor 1 and controls the proapoptotic *mTOR signaling* (Shoshani et al., 2002; Brugarolas et al., 2004). Also in the present study, the mRNA levels of two *DDIT4* isoforms increased in all investigated tissues, concurrently with modulated *mTOR signaling*. The modulation of the *DDIT4* expression across several tissues results most likely from glucocorticoids circulating in stressed individuals (Wong et al., 2010).

In summary, our study demonstrates that an acute temperature rise induces a significantly smaller set of DE genes in the liver, spleen and kidney of maraena whitefish than does a gradual temperature rise. The temperature-responsive genes commonly regulated in the respective tissues of whitefish may represent interesting candidates for future biomarker studies, as supported by several scientific reports about their functional involvement in stress-coping mechanisms across different animal models and experimental challenges.

## Conclusions

Stress has been defined as “a condition that disturbs the normal function of the biological system” (Sørensen et al., 2003). Vertebrates have developed different strategies to launch finely tuned responses for coping with different types of stressors. The present study investigated the influence of both acute and gradual change in ambient water temperature on maraena whitefish up to a critical temperature of 24°C. We clearly observed distinct transcriptomic responses to both kinds of thermal stress. In the three stress-relevant tissues (liver, spleen and kidney), 3 genes were commonly upregulated (*SERPINH1, EEF1A1, CLU*) and 8 genes were commonly downregulated (*EDF1, CEACAM20, CIRBP, AGT, YBX2, DDX5, SPCS2, FKBP5*) after gradual temperature rise. However, we noticed that the collection of genes responding to “gradual temperature rise” identified for other salmonid fish species so far is rather diverse and obviously strictly dependent on the duration of the experiment and the sampling time point, as well as on the chosen temperature range, the species and the tissues or cell types investigated. In contrast, the list of 11 genes responding to “acute temperature rise” (*HSP1A1, HSP90AA1, DDIT4, DNAJA4, PPP1R15A, DUSP1, DNAJB1, FOSL2, AHSA1, ZFAND2A*, and -*B*) that were exclusively upregulated after heat shock, is mostly in accordance with previous reports on acute-stress genes in vertebrates and specifically in salmonid fish, although rainbow trout and maraena whitefish, for instance, are at different stages of domestication and hence differentially adapted to anthropogenic environments. Subsequent analyses are required to evaluate if the identified list of DE genes constitutes a suitable set of biomarkers allowing for diagnostic predictions about the welfare status of whitefish in aquaculture facilities.

## Author contributions

TG, SA, and AR designed research. RB organized the production of fish and provided experimental facilities. MN, SA, MV, AR, and TG sampled fish and prepared samples for hybridization experiments. MN performed measurement of plasma parameters. AR and MN conducted RT-qPCR assays. AR and MV analyzed data. AR wrote the paper.

### Conflict of interest statement

The authors declare that the research was conducted in the absence of any commercial or financial relationships that could be construed as a potential conflict of interest. The reviewer SFC and handling Editor declared their shared affiliation.
