# Supplementary material for: Gradual and Acute Temperature Rise Induces Crossing Endocrine, Metabolic, and Immunological Pathways in Maraena Whitefish (Coregonus maraena)
Source: Front Genet. 2018 Jul 19;9:241. doi: 10.3389/fgene.2018.00241 (PMC6060367; doi:10.3389/fgene.2018.00241)
Supplement: Supplementary file 1 [file Image_1.PDF]

Supplementary Material

Gradual and acute temperature rise induce crossing endocrine, metabolic and immunological pathways in maraena whitefish (*Coregonus maraena*)

Alexander Rebl, Marieke Verleih, Mareen Nipkow, Simone Altmann, Ralf Bochert, Tom Goldammer*

*** Correspondence:** Tom Goldammer; tom.goldammer@fbn-dummerstorf.de

1.1 Supplementary Figures

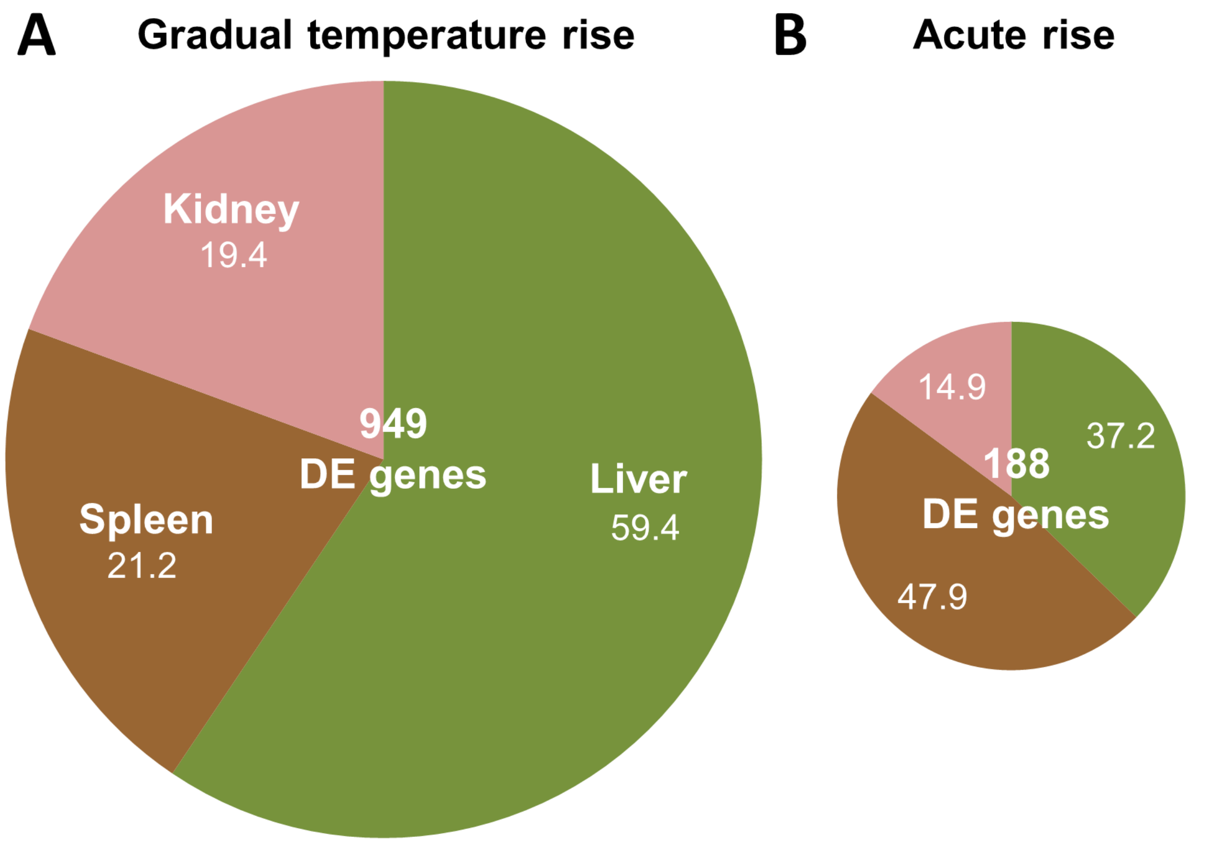

Supplementary Figure 1. Pie charts illustrate the percentage of the entirety of DE genes by liver (green circles), spleen (brown), and kidney (red) of maraena whitefish exposed to (**A**) gradual temperature rise and (**B**) acute temperature rise.

1.2 Supplementary Tables

Supplementary Table 1. QPCR-based verification of 10 selected genes identified as differentially expressed in a previous microarray analysis (*cf*. Fig. 4D). FC values result from the ratio of individuals belonging to the "gradual temperature rise" (grad) or "acute temperature rise" (acute) *versus* "temperature reference" (ref) groups.

**Tissue**	**Compa-rison**	**Fold-change values (qPCR)**									
		SERPINH1a	*HSPA1Aa*	*DDIT4a*	*DNAJA4*	*PPP1R15A*	*DUSP1*	*FOSL2*	*AHSA1*	*RGN*	*HP*
Liver	Grad/ref	**3.4****	**5.1**	1.7	**2.5** (*)	**0.3**	**0.4**	**0.3***	0.8	**5.6**	**0.3****
Liver	Acute/ref	**3.3***	**425.0***	**23.4** (*)	**44.8****	**3.1**	**9.1****	**8.6***	**2.8****	1.9	0.8
Spleen	Grad/ref	**8.3*****	**3.0***	0.8	**2.7***	1.5	**0.5**	1.2	1.6**	0.9	**0.4**
Spleen	Acute/ref	**4.1*****	**41.7*****	**7.0***	**5.1*****	**2.7****	**4.9*****	**3.4*****	1.7*	1.0	**0.2***
Kidney	Grad/ref	**5.3***	**2.7** (*)	0.6	1.9	0.6	0.8	1.0	1.0	0.9	0.8
Kidney	Acute/ref	**2.2****	**94.8****	**6.8****	**8.3*****	**3.1***	**4.4****	**2.4***	1.8***	**0.5**	0.8

Statistical significances are indicated with asterisks:

***, $p \leq 0.001$

**, $p \leq 0.01$

*, $p \leq 0.05$

(*), $p < 0.1$

FC > |2| is printed in bold face
